# The physico-chemical properties and structural characteristics of artificial soil for cut slope restoration in Southwestern China

**DOI:** 10.1038/srep20565

**Published:** 2016-02-17

**Authors:** Shunan Chen, Xiaoyan Ai, Tengyun Dong, Binbin Li, Ruihong Luo, Yingwei Ai, Zhaoqiong Chen, Chuanren Li

**Affiliations:** 1 Key Laboratory of Bio-resources and Eco-environment (Ministry of Education), College of Life Sciences, Sichuan University, Chengdu, Sichuan 610064, China; 2 College of Life Sciences, Sichuan Normal University, Chengdu, Sichuan 610066, China; 3 Chengdu medical college, Chengdu, Sichuan 610500, PR China

## Abstract

Cut slopes are frequently generated by construction work in hilly areas, and artificial soil is often sprayed onto them to promote ecological rehabilitation. The artificial soil properties are very important for effective management of the slopes. This paper uses fractal and moment methods to characterize soil particle size distribution (PSD) and aggregates composition. The fractal dimension (D) showed linear relationships between clay, silt, and sand contents, with coefficients of determination from 0.843 to 0.875, suggesting that using of D to evaluate the PSD of artificial soils is reasonable. The bias (*C*_*S*_) and peak convex (*C*_*E*_) coefficients showed significant correlations with structure failure rate, moisture content, and total porosity, which validated the moment method to quantitatively describe soil structure. Railway slope (RS) soil has lower organic carbon and soil moisture, and higher pH than natural slope soil. Overall, RS exhibited poor soil structure and physicochemical properties, increasing the risk of soil erosion. Hence, more effective management measures should be adopted to promote the restoration of cut slopes.

Large numbers of cut slopes are produced in the process of building transport infrastructure, which have severe impact on ecosystems. Soil properties at cut slopes are changed, since the topsoil and vegetation were destroyed. Natural succession processes are time consuming, and significant soil erosion, combined with adverse soil properties, make revegetation difficult. Hence, measuring the slope characteristics is essential when considering slope stability, environmental protection, and aesthetics, to promote vegetation restoration^1^,^2^,^3^. Numerous techniques have been attempted to provide an ecologically sustainable and socio-economic method for restoring cut slopes^4^,^5^,^6^. Hydro-seeding, for example, sprays an artificial soil mixture onto cut slopes to supply root anchorage and nutrient sources for vegetation. The depth of the artificial soil is approximately 10 cm, and the backfill soil is an important component of the artificial mixture. Restoration practitioners have begun to use rock fragments obtained from the cut slope surface after crushing and passing through 2 mm sieves to replace agricultural soil as backfill. This method is commonly used to restore cut slopes in southwest China, because it is very convenient and economical. However, rock fragments have not undergone adequate soil forming processes, and it is unclear if this contributes to establishing vegetation and slope stability. Hence, the edaphic characteristics of this artificial system must be evaluated and monitored.

Soil quality influences its basic functions, such as retaining water, promoting biodiversity, supporting agriculture, and resisting flooding, erosion, and landslides^7^. Maintenance of soil quality is critical for ensuring the sustainability of the environment and the biosphere. Previous soil research has been largely concentrated on agricultural^8^,^9^,^10^, forest^11^,^12^,^13^, and typical grassland^14^,^15^ soils. Recently, ecological restoration has been widely discussed with increasing awareness of environmental conservation issues, and considerable documentation produced regarding slope soil, particularly slope restoration and stabilization^16^,^17^,^18^. Katz *et al.* (2011) used numerical modeling to assess rock fall hazards and associated risk in the Soreq and Refaim valleys, and showed that rock falls induced by earthquakes can damage the road network. Chirico *et al.* (2013), amongst others, examined the role of vegetation in the unsaturated region for stability of shallow soils, and found that vegetation helps to control soil loss and stabilize cut slopes, because vegetation soil systems enhance the soil shear strength^19^,^20^. However, recent studies have shown that vegetation degeneration and soil erosion may occur several years after artificial revegetation^21^,^22^,^23^. To achieve successful long term restoration of cut slopes, artificial soil must be studied and understood.

Railways are the most effective overland transport in China, and the protection of rock cut slopes during railway construction is very important for local railroad safety^24^. However, there is little available information about the edaphic characteristics of this artificial system. In this study, the physicochemical characteristics of artificial soil were measured, as well as their interrelationships. Soil properties, such as bulk density, porosity, pH value, cationic exchange capacity (CEC), soil organic carbon (SOC), and moisture content are closely related to soil quality. We chose edaphic properties (physical, chemical, and biochemical properties) to assess soil quality. The combined selected indicators reflect the physical and chemical characteristics of artificial soil. Our main objectives were to better understand the characteristics of artificial soil on rock cut slopes through chemical and physical characteristics, to assess the sustainability of artificial soil applied to cut slopes, and provide a theoretical and practical basis for artificial soil restoration.

## Materials and Methods

### Study site

The study site was located in the vicinity of Suining railway station, Suining, Sichuan Province, China (lat. 30°32′N, long. 105°32′E). This area has humid subtropical monsoonal climate, mean annual temperature 17.4 °C, average annual precipitation 927.6 mm, and average annual wind velocity 0.7 m/s. The soil at the study site was classified as Eutric Cambisol using the FAO-UNESCO system. Geographically, the area is characterized by uneven topographic profile with many hills, and many cut slopes formed during railway construction and subsequently hydro-seeded. Artificial soil used in hydro-seeding included rock fragments obtained from the cut slope surface.

**Figure 1:**
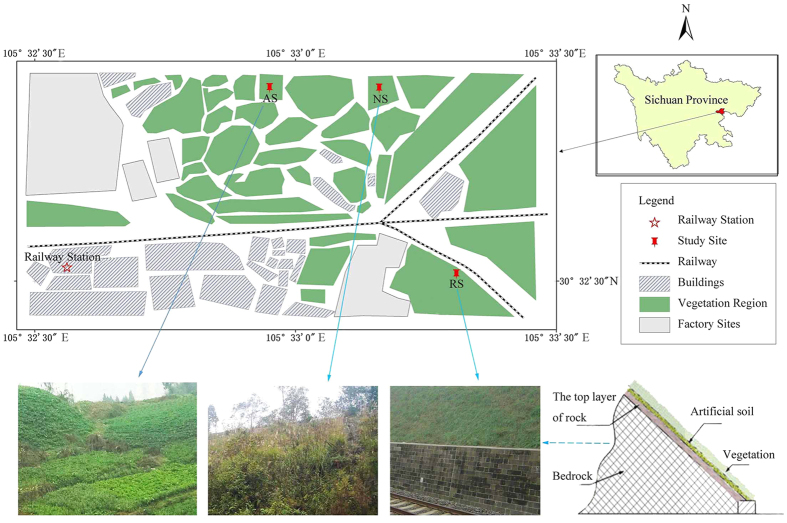
Detailed locations of the study sites. Notes: RS = railway slope; NS = a naturally developed slope within 1 km of the railway; AS = an agricultural slope within 1 km of the railway. This map was generated by the authors by using the ArcGIS (version10.0). The photographs in this map was taken by one of the authors (Shunan Chen).

We selected three similar slopes with different land uses, as shown in Fig. 1:

1. Rock-cut slope (RS). Excavated for railway construction in 2003, and hydro-seeded in September 2004;
2. Natural slope (NS) without human disturbance;
3. Agricultural slope (AS) under conventional tillage.

RS was the main study object, with NS and AS providing references. All the slopes are approximately 45 m high with approximately 40° gradient.

### Sample collection and determination

Data collection was by randomized sampling followed an experimental design with three treatments and four replicates per treatment. Twenty-five actual soil samples comprised an individual analysis sample, from which four duplicate samples were drawn and fully mixed before analysis. The samples were obtained from 5–8 cm beneath the soil surface, as the backfill soil cover was less than 10 cm. Soil moisture and bulk density were determined immediately after sampling using the oven dry and cutting ring methods, respectively. After plant tissues and gravel were removed, soil samples were air dried in the laboratory. Aggregate stability was measured by Yoder’s method, incorporating dry and wet sieving^25^. Small particles percentages (<0.002, 0.002–0.02, 0.02–0.05, and 0.05–0.25 mm) were measured by the pipette method^26^. Each air dried sample was finely ground and passed through a 1 mm sieve then measured for total porosity, CEC, SOC, and pH^27^,^28^,^29^. The activities of Catalase, Urease, and Sucrase were measured following Singh *et al.*^30^. Microbial biomass C and N were measured following Xue *et al.*^31^.

### Determination of soil structure parameters

Soil textural class was identified following the ISSS soil texture classification system. The proportions of clay (<0.002 mm), silt (0.002–0.02 mm), and sand (0.02–2 mm) were used to characterize particle size distribution (PSD). The fractal dimension (D) was used to express mass and size information about soil particles^26^,^32^,


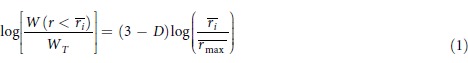


where *W* is the cumulative mass (g) of particles with diameter less than 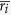; *W*_*T*_ is the total mass (g) of the particles; 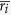 is the mean particle diameter (mm) of the *i*th size class, i.e., the arithmetic mean of the upper and lower sieve sizes; and 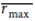 is the mean diameter (mm) of the largest size class.

The moment method was used to evaluate soil aggregate size distribution, because the bias (C_*S*_) and peak convex (C_*E*_) coefficients reflect the symmetry and concave and convex features of the frequency distribution curves for soil aggregates^33^,^34^,


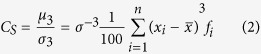



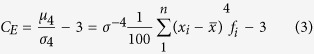


where *f*_*i*_ is the weight ratio of the aggregate remaining on the sieve; *σ* is the standard deviation; *C*_*S*_ is the bias coefficient, indicating symmetry of frequency distribution curves of soil aggregates; and *C*_*E*_is the peak convex coefficient, indicating the convexity of the frequency distribution curve for soil aggregates. When *C*_*S*_ > 0, larger aggregates are numerically dominant; whereas when *C*_*S*_ < 0, smaller aggregates are numerically dominant. When *C*_*E*_ > 0, the aggregates size distribution is relatively concentrated in a small range; whereas when *C*_*E*_ < 0, the aggregates size distribution covers a wider range.

The destruction rate of soil aggregates (P) is an important index for evaluating erosion durability, with higher P values implying lower soil aggregate stability,


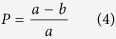


where *a* is the percentage of aggregates >0.25 mm, measured by Yoder’s method, dry sieving; and *b* is the percentage of water stable aggregates >0.25 mm, measured by Yoder’s method, wet sieving.

### Statistical analysis

One-way analysis of variance was used to compare soil properties among the slope patterns, Fisher’s least significant difference (LSD) was used for the mean separation at 0.05 0.01 significance, Spearman’s test was used to determine correlations between soil properties, and linear regression was used for the PSD fractal dimensions. All analyses were conducted using SPSS software (19.0).

## Results and Discussion

### Soil texture and fractal features

Table 1 shows the relevant measurement and analysis results. There are considerable differences in PSD between the three land uses. RS has the lowest clay and highest sand content, while NS has the highest clay and lowest sand content. For all land uses, the dominant soil particle is sand (59%–85%). RS texture is loamy sand, whereas NS and AS are sandy loam, i.e., RS texture is coarsening, while AS remains similar to NS. Hence, NS has experienced sufficient soil forming processes without human disturbance, contributing to the soil formation and stability. NS stable structure enhances finer particle fractions, providing the surface with high resistance to runoff in the rainy season. Although AS has similar texture to NS, considering sand content only, AS has higher course particles content than NS because AS aggregates had been broken down by tillage, generating a larger amount of small and more easily transportable particles and micro-aggregates^21^. RS is largely composed of dispersed rock particles and has not undergone adequate soil formation processes. This artificial soil sprayed onto the bare rock slopes had little viscidity with the rock surface, and became more susceptible to erosion after rainfall, which may have caused the fine particles to be removed, leaving the coarser ones, and indirectly led to the lack of water and nutrient supply for plants on the slope. RS texture is harsher than NS for slope revegetation. Consequently, two soil improvement treatments should be actioned: increase the viscidity between the artificial soil and rock, and improve the long term availability of nutrients and water.

The PSD fractal dimension (D) ranges from 2.61 (RS) to 2.72 (NS), and the differences among the three different land uses are significant (Table 1). The variations in the trend of D are consistent with the soil’s clay and silt contents. The fractal dimension of RS is significantly lower AS, indicating that RS has lost micro-particles and is coarsening. Thus, even with the addition of soil supplements, such as humus and water retention agents, RS has not undergone sufficient long term soil forming processes and is composed of dispersed rock particles rather than soil aggregates. The unstable RS structure leads to the loss of the finer particles, especially on high gradient slopes, where surface runoff is frequently intense in the rainy season, and this tendency is reflected in lower D values. AS has undergone adequate field management and soil improvement treatments, and so has proportionally more micro-particles. The fractal dimension of NS is significantly larger than AS, because the removal of human disturbance (tillage), is advantageous for forming favorable soil structurers, retaining finer particles and enhancing the fractal dimension.

**Figure 2:**
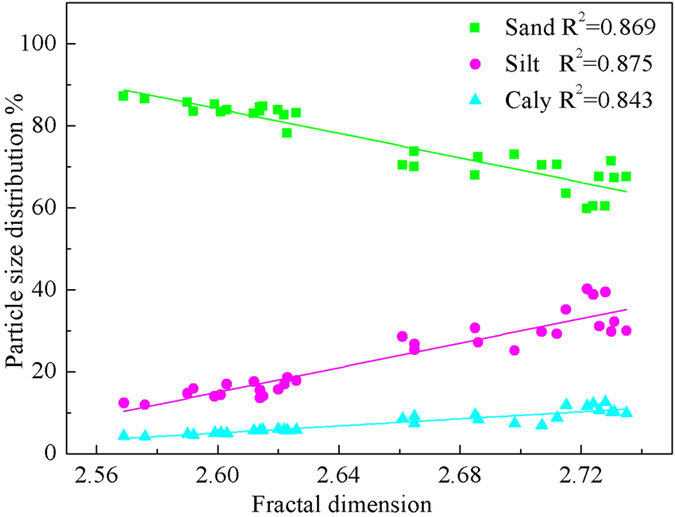
Linear regression for fractal dimension and particle size distribution. Notes: ■ Sand, y = 473.059 – 149.568 x; • Silt, y = −374.228 + 149.708 x; ▲ Clay, y = −106.984 + 43.105 x.

Figure 2 shows that D has a positive correlation with fine particle content, i.e., D increases as clay and silt content increases. However, D correlates negatively with course particle content, i.e., D decreases as sand content increases. The coefficients of determination for the linear regressions range from 0.843 to 0.875, indicating that it is reasonable to use fractal models to evaluate the PSD of this artificial backfill soil.

### Physical chemical properties of artificial soil

Physical chemical properties of the soil, such as bulk density, porosity, pH, and soil organic carbon, summarized in Table 2, are usually considered as indicators of soil quality^35^,^36^. The three soils are slightly alkaline, with pH from 7.83 to 7.94. NS has significantly lower soil pH than RS and AS, which do not differ significantly. Particle repulsion clay particles has been shown to increase with increasing pH^37^. Therefore, controlling soil pH is very important in controlling the dispersion of clay within the soil. Table 1 shows significant differences of clay fractions among the three land-use patterns, which may account for differences in pH of these soil. Table 3 shows that soil pH is negatively correlated with other soil indicators, except for structure failure rate. In particular, pH is significantly negatively correlated with SOC. Table 2 shows significantly differences in CEC among the three land uses, with NS > AS > RS. Low CEC implies the soil is able to hold less nutrients applied through fertilization^38^. Hence, since RS has low CEC, the availability of nutrients to plants is limited. Spearman’s correlation (Table 3) shows CEC is positively correlated with D, moisture content, and SOC, and negatively correlated with pH. Therefore, decreasing the artificial soil pH will provide a more suitable environment for revegetation when using rock fragments as backfill onto the slope surface.

Table 2 shows the soil moisture content of RS is lowest among the three land uses. Soil moisture regimes can be modified by management practices, such as irrigation, cover cropping, and mulching, hence AS moisture content is significantly higher than RS. Soil structure and texture affects water flow, availability, and storage. High large particle content increases pore spaces and reduces capillary porosity, decreasing retention and availability of moisture through evaporation and infiltration^39^. RS structure is poor and coarsening, and consequently the water holding capacity is poor. Thus, RS moisture is significantly less than the other two land uses. Some studies have shown that changes in SOC associated with smaller soil particle (<0.25 mm) fractions may reflect changes in soil management^40^. Table 2 shows significant differences in SOC content among the three land uses, with NS > AS > RS, which may be a consequence of their relative PSDs. Micro-aggregates can protect SOC decomposition because their small pore effectively limits bacterial access^41^. AS has higher SOC than RS, which may be because AS has been supplemented with fertilizers containing organic matter. In contrast, NS has the highest SOC content, and, therefore, provides more nutrients for plants growth and is more suitable for restoration and succession of slope vegetation.

Soil bulk density also shows significantly differences among the three land uses (Table 2) with RS > NS > AS. However, the total porosity trend is opposite, with RS < NS < AS. RS is composed of dispersed rock particles, with the dominant soil particle being sand. Sand particles pack together best, producing lowest total porosity and highest bulk density. The finer particles of AS produce greater porosity and lower bulk density due to tillage. However, tillage can only produce a short term increase in soil porosity, rather than long term decrease in soil aggregation. Some research has shown that SOC can enhance soil resistance through several mechanisms (strengthening of binding forces among particles and aggregates, and increasing elasticity of aggregates under compression) and produce higher porosity and lower soil bulk density^42^. Soil organic carbon is an important factor affecting the formation of soil structure. Table 2 shows RS has the lowest SOC content, and shows a similar trend across all the biochemical parameters, as shown in Table 4, where NS > AS > RS for all parameters, and are all significantly different, except Sucrase. These confirm that RS quality is poor, the ecological environment is harsh and biological activity is low. RS nutrients available to vegetation is less than NS, and is not conducive to plant growth. Applying biochar can improve the biological properties^43^, so adding organic fertilizer and biochar to RS will promote ecological restoration, but further research is needed regarding practical application methods.

### Stability of soil aggregates and aggregate size distribution

The status of soil dry aggregates and water-stable aggregates not only reflect soil structure, but also soil fertility^44^. Table 5 shows significantly differences in the water stable aggregates (>0.25 mm) among the three land uses. Aggregate content of NS is significantly higher than AS and RS, from both dry and wet sieving. The water stable aggregate content of RS is significantly higher than AS, with AS < RS < NS. There are no significant differences between dry aggregates (>0.25 mm) for RS and AS. Thus, large aggregate content (>0.25 mm) in AS and RS are similar, without rain washing, whereas water stable aggregate content is significantly different. RS shows greater soil destruction rates of soil aggregates than NS, with AS > RS > NS. This may be due to human disturbance and the absence of effective protection causing structure degradation, and clay and organic matter loss, so soil aggregates collapse more easily, and consequently produce higher destruction rates.

**Figure 3:**
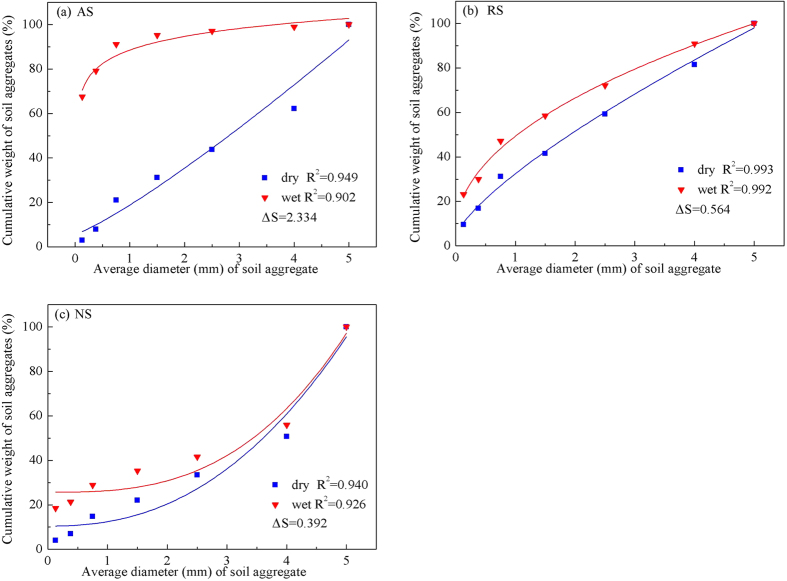
Cumulative distribution of soil aggregate weights after dry and wet sieving. Notes: (**a**) AS = Agricultural slope; (**b**) RS = rock-cut slope; (**c**) NS = natural slope. ∆S = area difference between the dry and wet sieve aggregate cumulative distributions.

Soil aggregate stability an important factor for soil structure. Figure 2 shows the cumulative distribution of soil aggregate weights after dry and wet sieving for the three land-uses. The area difference (∆S) between the dry and wet sieved curves reflects aggregate stability (Fig. 3), where stability is decreased for larger areas, the area differences (Fig. 3) ranged from 2.334 (AS) to 0.392 (NS), i.e., aggregate stability of AS is significantly less than NS. This is consistent with the structure failure rate (Table 5), which follow NS > RS > AS. NS has significantly more aggregate stability than RS. RS is artificial rock fragments with additional supplements, such as humus and water retention agents, but its eluviation, deposition, transport, and biological cycles are acutely deficient, which make it vulnerable to rainfall and wind erosion. Soil erosion resistance weaker composition of the material, is caused by soil erosion particularly serious underlying causes^45^. We suggest using soil that has undergone sufficient long term soil forming processes for repairing cut slopes, to increase soil erosion resistance and reduce the risk of slope protection failure.

Table 5 shows the characteristic parameters the for soil aggregate size distributions. There are significant differences among the three land uses. *C*_*S*_ decreases from NS to AS, with AS being negative. A similar trend is evident for *C*_*E*_. This shows NS has good aggregate stability, resistance to dispersion, and larger aggregates have numerical advantage. RS has weaker aggregate stability, aggregate size is smaller, and resistance to mechanical crushing was poor. This may be because the soil sprayed onto rock cut slopes has little viscidity on the rocks and/or lack of water and nutrient supply for plants. Therefore, RS has poorer soil erosion resistance than NS. Long term continuous cultivation can degrade soil due to erosion, organic matter loss, and destruction of soil structure. Hence, AS aggregate stability is poorer NS. *C*_*S*_ and *C*_*E*_ both show significant negative correlations with structure failure rate (R = −0.992 and R = −0.994, respectively, Table 3), and significant correlation with moisture content and total porosity. Thus, *C*_*S*_ and *C*_*E*_ can be used to quantitatively describe soil structure. The moment method utilizes all information about aggregate analysis, and may be a useful tool to evaluate soil structure development during slope restoration.

## Conclusions

This study investigated the physical chemical properties of artificial soil on cut slopes formed during railroad construction.

RS shows poorer quality characterized by significant loss of fine particles; lower soil moisture, CEC, and organic carbon content; weaker structural stability; higher soil structure failure and pH, compared with NS. Thus, RS texture was harsh for slope revegetation and has high risk of surface runoff and soil erosion due to insufficient soil forming processes.

More effective protection measures should be taken to promote restoration of cut slopes: Increase viscidity between artificial soil and rock surfaces to reduce soil erosion and the risk of slope protection failure; decrease artificial soil pH with to provide a more suitable soil environment for ecological restoration; and increase soil nutrients available to vegetation by adding organic fertilizer and biochar. Further research is needed regarding practical application methods.

Fractal dimension is sensitive to the increased coarse particle content, is linearly related to selected physical chemical properties of the soil. On the other hand, the moment method utilizes all the soil aggregate information. Therefore, the fractal and moment methods can be considered appropriate indexes to evaluate overall quality of synthetic soil used for revegetation of a cut slope.

This study has highlighted that poor physical chemical properties of artificial soil lack the necessary conditions for vegetation recovery and the slope surface lacks supporting stability. These will cause landslides, with consequential impacts on transportation and safety. Ecological rehabilitation of railway slopes is imperative. Future study should extend this work to provide better understanding of the requirements for artificial soil used in ecological restoration of cut slopes.

## Additional Information

**How to cite this article**: Chen, S. *et al.* The physico-chemical properties and structural characteristics of artificial soil for cut slope restoration in Southwestern China. *Sci. Rep.* **6**, 20565; doi: 10.1038/srep20565 (2016).

## Figures and Tables

**Table 1:** Soil texture and particle size distribution.

Slope patterns	PSD			Textural class	Fractal dimension
	Sand %	Silt %	Clay %		
RS	85.94 ± 1.31^a^	13.68 ± 1.21^c^	4.93 ± 0.72^c^	Loamy Sand	2.61 ± 0.01^c^
NS	59.53 ± 0.98^c^	42.85 ± 0.87^a^	12.14 ± 0.48^a^	Sandy Loam	2.72 ± 0.003^a^
AS	71.46 ± 4.16^b^	26.91 ± 4.61^b^	9.47 ± 1.10^b^	Sandy Loam	2.70 ± 0.02^b^
*F* value	78.89**	81.65**	61.14**		113.35**

Notes: RS = cut slopes reconstructed from rock fragments. AS = agricultural slope. NS = natural slopes. Values in each column with the same letter are not significantly different (p > 0.05, LSD) among the slope patterns. **Significant at the 0.01 level.

**Table 2:** Fundamental physical and chemical properties of slope soils.

Slope patterns	pH	SOC (g•kg^−1^)	Moisture content (%)	Bulk density (g•cm^3^)	Total porosity (%)	CEC (cmol^+^kg^−1^)
RS	7.94 ± 0.02^a^	16.68 ± 0.57^c^	8.12 ± 2.00^b^	1.46 ± 0.07^a^	46.15 ± 2.78^c^	9.49 ± 0.23^c^
NS	7.83 ± 0.01^b^	22.80 ± 0.49^a^	19.65 ± 0.89^a^	1.28 ± 0.04^b^	52.52 ± 1.96^b^	13.31 ± 0.13^a^
AS	7.92 ± 0.01^a^	21.56 ± 0.85^b^	21.80 ± 1.48^a^	1.10 ± 0.05^c^	60.08 ± 1.56^a^	12.30 ± 0.35^b^
*F* value	77.62**	73.99**	77.42**	30.07**	31.13**	188.02**

Notes: SOC = soil organic carbon. CEC = cation exchange capacity. Values in each column with the same letter are not significantly different (p > 0.05, LSD) among the slope patterns. **Significant at the 0.01 level.

**Table 3:** Pearson correlation coefficients for characteristic parameters and selected soil properties.

	pH	D	P	*C*_*S*_	*C*_*E*_	MC	BD	SOC	CEC
D	−0.499								
P	0.474	0.509							
*C*_*S*_	−0.540	−0.437	−0.992**						
*C*_*E*_	−0.488	−0.500	−0.994**	0.991**					
MC	−0.553	0.969**	0.441	0.364	−0.440				
BD	−0.209	−0.883**	−0.712*	0.648*	0.700*	−0.894**			
SOC	−0.755**	0.885**	0.131	−0.049	−0.131	0.912**	−0.687*		
CEC	−0.817**	0.876**	0.172	0.012	−0.061	0.892**	−0.648*	0.988**	
TP	−0.165	0.866**	0.741*	−0.679*	−0.731*	0.874**	−0.997**	0.655*	0.612*

Notes: P = structure failure rate (%). D = Fractal dimension. SOC = soil organic carbon (g•kg^**−**1^). MC = moisture content (%). BD = bulk density (g•cm^3^). TP = total porosity (%). CEC = cation exchange capacity (cmol^+^kg^**−**1^). Significant differences, *P < 0.05 and **P < 0.01.

**Table 4:** Biochemical parameters of slope soils.

Slope patterns	Catalase (ml•g^−1^)	Urease (mg•g^−1^)	Sucrase (mg•g^−1^)	Microbial biomass C (mg•kg^−1^)	Microbial biomass N (mg•kg^−1^)
RS	1.42 ± 0.04^c^	31.23 ± 0.95^c^	1.76 ± 0.15^b^	135.94 ± 4.67^c^	27.77 ± 1.90^c^
NS	2.01 ± 0.08^a^	41.05 ± 0.41^a^	8.29 ± 0.57^a^	194.03 ± 5.12^a^	54.65 ± 1.17^a^
AS	1.71 ± 0.03^b^	38.39 ± 1.40^b^	7.19 ± 0.93^a^	176.44 ± 1.48^b^	45.79 ± 2.62^b^
F value	112.17**	76.41**	89.91**	159.24**	166.96**

Notes: Values in each column with the same letter are not significantly different (p > 0.05, LSD) among the slope patterns. Significant differences, *P < 0.05 and **P < 0.01.

**Table 5:** Aggregate stability and characteristic parameters of size distribution.

Slope patterns	Sieving method	>0.25 mm (%)	Structure failure rate (P) (%)	*C*_*S*_	*C*_*E*_
RS	Dry-sieving method	90.65 ± 0.74^b^	28.06 ± 2.23^b^	0.95 ± 0.11^b^	0.52 ± 0.08^b^
	Wet-sieving method	65.21 ± 1.77^b^			
	Dry-sieving method	96.46 ± 0.53^a^			
NS	Wet-sieving method	77.82 ± 0.99^a^	19.32 ± 1.29^c^	1.49 ± 0.06^a^	0.86 ± 0.04^a^
	Dry-sieving method	90.16 ± 2.06^b^			
AS	Wet-sieving method	33.67 ± 1.31^c^	62.61 ± 2.17^a^	−0.39 ± 0.04^c^	−0.66 ± 0.05^c^
		21.70^**^			
*F* value		796.04**	416.21**	510.94**	575.31**

Notes: C_S_ = bias coefficient. C_E_ = peak convex coefficient. Values in each column with the same letter are not significantly different (p > 0.05, LSD) among the slope patterns. **Significant at the 0.01 level.
